# Association of Plasma Transforming Growth Factor-β1 Levels and the Risk of Atrial Fibrillation: A Meta-Analysis

**DOI:** 10.1371/journal.pone.0155275

**Published:** 2016-05-12

**Authors:** Jiao Li, Yajuan Yang, Chee Yuan Ng, Zhiwei Zhang, Tong Liu, Guangping Li

**Affiliations:** 1 Tianjin Key Laboratory of Ionic-Molecular Function of Cardiovascular disease, Department of Cardiology, Tianjin Institute of Cardiology, Second Hospital of Tianjin Medical University, Tianjin, 300211, People’s Republic of China; 2 Cardiac Arrhythmia Service, Massachusetts General Hospital, 55 Fruit St., Boston, Massachusetts, 02114, United States of America; The Ohio State University, UNITED STATES

## Abstract

**Introduction:**

Numerous studies have demonstrated that plasma transforming growth factor-β1 (TGF-β1) may be involved in the pathogenesis of atrial fibrillation (AF), but some discrepancy remained. We performed a meta-analysis to evaluate the association between the plasma level of TGF-β1 and the risk of AF.

**Methods:**

Published clinical studies evaluating the association between the plasma level of TGF-β1 and the risk of AF were retrieved from PubMed and EMBASE databases. Two reviewers independently evaluated the quality of the included studies and extracted study data. Subgroup analysis and sensitivity analysis were performed to evaluate for heterogeneity between studies.

**Results:**

Of the 395 studies identified initially, 13 studies were included into our analysis, with a total of 3354 patients. Higher plasma level of TGF-β1 was associated with increased risk of AF when evaluated as both a continuous variable (SMD 0.67; 95%CI 0.29–1.05) and a categorical variable (OR 1.01, 95% CI 1.01–1.02).

**Conclusions:**

This meta-analysis suggests an association between elevated plasma TGF-β1 and new onset AF. Additional studies with larger sample sizes are needed to further investigate the relationship between plasma TGF-β1 and the occurrence of AF.

## Introduction

Atrial fibrillation (AF) is the most common sustained arrhythmia with debilitating consequences such as stroke and heart failure. It is also associated with an increase in overall mortality [1,2,3]. Animal models and studies on patients with AF have confirmed that the development of AF is associated with both structural and electrical remodeling of the atria [4]. Patients with chronic AF have significant myocardial interstitial fibrosis which contributes to the occurrence and perpetuation of AF [5, 6]. Transforming growth factor-β1 (TGF-β1) is an important factor in fibrosis [7]. It is involved in the process of cell proliferation, apoptosis and migration. It promotes the differentiation of cardiac fibroblasts and production of extracellular matrix such as collagen, fibronectin, and protein polysaccharide which leads to cardiac fibrosis [8]. In transgenic mouse models, the activation of TGF-β1 promotes atrial fibrosis and the development of AF [9]. On the other hand, the inhibition of TGF-β1 by pirfenidone (PFD) can significantly reduce the extent of atrial fibrosis [10]. These findings have prompted clinical studies on the relationship between plasma TGF-β1 levels and the development of AF in humans. However, the results generated have been inconsistent. Therefore, we conducted a comprehensive meta-analysis to evaluate the available evidence of whether high plasma TGF-β1 levels are related to the risk of having AF.

## Methods

### Search strategy

Articles were identified by searching PubMed and Embase online databases for articles published up until November 2015. The key terms used are ‘TGF-β1’, ‘transforming growth factor-β1’, ‘transforming growth factor-beta1’, ‘transforming growth factor’ and ‘atrial fibrillation’. We manually searched the bibliographies of original papers and abstracts of the scientific sessions of the past 3 years. In addition, we sought the assistance from potential experts in the field to assess the quality of included articles. We evaluated the titles, abstracts and reference lists of all articles to identify potentially relevant studies.

### Trial selection and inclusion criteria

Two reviewers (J. L. and Y. Y.) evaluated the titles and abstracts of all eligible studies. The full text of relevant studies was retrieved and assessed accordingly based on the inclusion criteria. Any disagreements on whether to include any study between the two investigators were resolved through joint review and discussions.

For inclusion, eligible trials should meet the following criteria: (1) the study design was case-control, prospective or retrospective cohort studies; (2) human subjects; (3) included the characteristics of study patients; (4) clearly defined endpoint events, such as AF occurrence or recurrence; (5) evaluated the plasma TGF-β1 levels of AF patients and non-AF patients; (6) reported the plasma level of TGF-β1 using [mean ± standard deviation (SD)] and odds ratio (OR) or hazard ratio (HR) of AF incidence and the corresponding 95% confidence interval (CI) for TGF-β1 levels.

### Data extraction

Two independent reviewers (J. L. and Y. Y.) extracted data from included studies using a standard data extraction form. Information on authors and published journals were removed and then independently evaluated according to the described inclusion criteria. Relevant data were extracted from the manuscripts. We extracted and analyzed the plasma concentration of TGF-β1 expressed as mean ± SD from each primary study. Adjusted OR values were selected for the analysis. Additional data collected included study characteristics (first author’s last name, publication year, study design, sample size, AF definition, follow-up duration, end-point events) and patients baseline characteristics (age, sex, BMI, smoking, mean left atria diameter, left ventricular ejection fraction, the presence of CAD, hypertension, diabetes and medication).

### Quality assessment

The two investigators (J. L. and Y. Y.) independently evaluated the quality of the eligible studies based on the guidelines by the Evidence-Based Medicine Working Group [11] and the United States Preventive Task Force [12]. Each study was judged in accordance to the 10-item STROBE checklist. We appraised the quality of studies according to the following characteristics: (1) the inclusion and exclusion criteria are clearly defined; (2) sample selection is clearly described; (3) involved population is representative of study sample; (4) the patients’ follow-up period is adequate; (5) reports loss of follow-up; (6) clinical and demographic variables are complete; (7) the definition of AF is clearly defined; (8) the outcomes and outcome assessment are clearly defined; (9) temporality (evaluation of plasma TGF-β1 levels at baseline) and (10) adjustment of possible confounders on the multivariate analysis, especially for categorical variable. If any of the characteristics was not described, we assumed that it had not been performed.

### Statistical analysis

All continuous variables were presented as (mean ± SD). The standard mean difference (SMD) was used to analyze the results in our meta-analysis. SMD method was used as different unit of measurements were presented for TGF-β1 levels. As the studies included in this meta-analysis may have used either a continuous or categorical variable for TGF-β1 levels, we performed a separate meta-analysis for both types of variables to evaluate the association between TGF-β1 levels and the occurrence of AF. The HR values in multivariate Cox proportional hazards model in each primary study were directly considered as OR values. I^2^ derived from the chi-square test was used to evaluate the heterogeneity across the studies included. I^2^ of ≤50% indicates that there was no significant heterogeneity [13]. A fixed effects model was used if no significant heterogeneity was found. When pooled effect resulted in significant heterogeneity, the random effects model was used. We conducted random effects meta-analysis using the inverse variance heterogeneity method. In addition, we also performed subgroup analysis based on the patients’ age (≤50y or >50y), study design (cohort study or case control study), duration of follow-up (<12 months or ≥12 months), sample size (<100 or ≥100) and left ventricular ejection fraction (LVEF) (<50% or ≥50%). Sensitivity analysis was performed by sequentially removing each individual study. We assessed for publication bias by constructing a funnel plot. Two-tailed p value of <0.05 was considered statistically significant. All statistical analyses were performed with Review Manager Version 5.3.

## Results

### Search results

**Fig 1:**
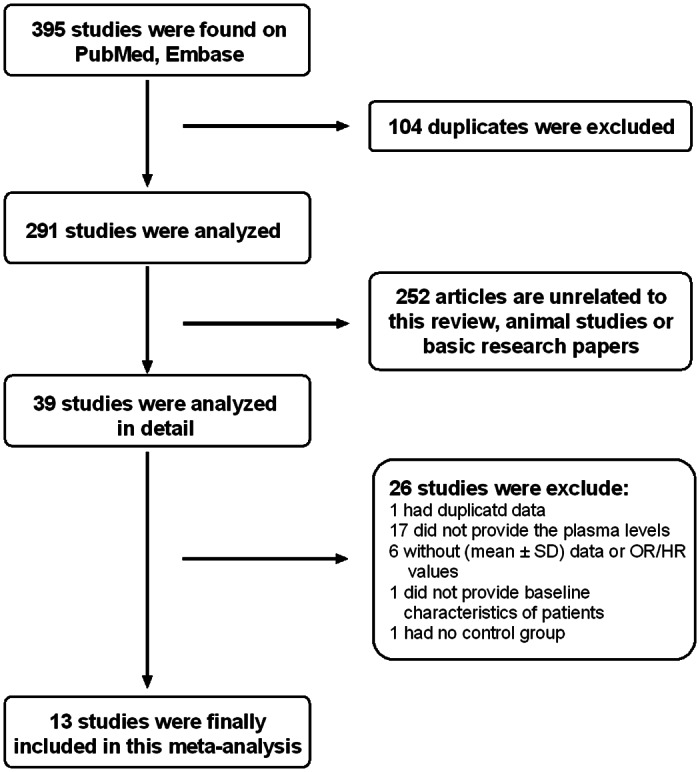
Flow chart of study selection. SD, standard deviation.

Data retrieval and study selection was shown in the flow chart (Fig 1). A total of 395 studies were found using our search criteria. After reviewing title and abstract of each study, we excluded 356 articles because they were either unrelated, review articles or basic science research papers. Then, we evaluated the remaining 39 studies in detail. Of these 39 studies, we excluded 26 studies because: 1 had duplicate data, 17 did not provide the plasma TGF-β1 levels, 6 did not provide (mean ± SD) data of TGF-β1 or OR/HR values, 1 did not provide baseline characteristics of patients and 1 had no control group. Finally, the remaining 13 studies were included into our meta-analysis.

### Study characteristics

We included 13 studies with a total of 3354 patients of which 1154 patients have AF and 2200 patients have no AF. The main features of the studies are exhibited in Table 1, and the patients’ baseline characteristics are summarized in Table 2. Confounding factors used in multivariate analysis are shown in Table 3. Out of the 13 included studies, 6 studies[14–19] demonstrated that AF patients had higher plasma TGF-β1 levels, regardless of whether it was new-onset AF or recurrent AF, but the remaining 7[20–26] presented no significant correlation between plasma TGF-β1 levels and occurrence of AF. 12[14–26] studies in which the plasma TGF-β1 levels was expressed as (mean ± SD) were analyzed using TGF- β1 as a continuous variable. 4[15, 19, 25, 26] studies with OR/HR values in which the plasma TGF-β1 levels was analyzed as a categorical variable had been included in a separate analysis. Of the 13 included studies, 3 studies [15, 19, 25] analyzed TGF-β1 levels as both a continuous variable and categorical variable.

**Table 1:** General data of studies included in meta-analysis.

Investigator (year)	Location	Patients number (n)	Study population	Design type	Mean follow-up	Endpoint	Duration of AF	Quality score
Wang 2010	China	540	Patients who were newly diagnosed essential hypertensive and none of them received anti-hypertensive treatment.	Case control study	During hospitalization	The occurrence of AF was determined by 12-lead electrocardiography (ECG) and/or 24-h Holter monitoring.	NA	7
Wu 2013	Taiwan	46	Nonparoxysmal AF patients who underwent catheter ablation.	Cohort study	10.9 ± 7.4 months	The clinically documented recurrence of atrial arrhythmias or repeat ablation procedures. An AF recurrence was defined as an episode lasting >1 minute and was confirmed by ECG 3 months after the ablation.	71.3±58.1 months	9
On 2009	Korea	76	Patients who underwent both the open heart operation for mitral valvular heart disease and the surgical maze procedure for AF.	Cohort study	12 months	The primary end point of the study was the persistence of AF after the maze procedure with cryoablation.	3.4 years	8
Xiao 2010	China	38	Patients with RHD who underwent valve replacement surgery.	Case control study	During hospitalization	The occurrence of AF. The patients were divided into 3 groups: the sinus rhythm group, the paroxysmal AF group, and the chronic AF group (AF lasting ≥6 months).	NA	7
Zhao 2014	China	90	VHD patients, comprising pathological changes in the mitral or aortic valves, or both, who underwent valve replacement surgery	Case control study	During hospitalization	The occurrence of AF. (Persistent AF:AF lasting >6 month and paroxysmal AF: recurrent AF that terminated spontaneously in <7 days.)	NA	7
Kim 2009	Korea	74	Patients with persistent AF who underwent external electrical cardioversion.	Cohort study	13.2 ± 11.0 months	AF recurrence after successful cardioversion.	NA	8
Mira 2013	China	80	Patients with AF.	Case control study	During hospitalization	The occurrence of AF. Patients were divided into paroxysmal AF group and persistent AF group according to whether they could convert to sinus rhythm spontaneously.	NA	7
Lin 2015	China	112	Patients with a history of essential hypertensive.	Case control study	During hospitalization	AF was determined by 12-lead electrocardiography (ECG) and/or 24-h Holter monitoring. Persistent AF: AF lasting >6 month.	13.12±9.96 years	7
Kimura 2014	Japan	44	AF patients who received an initial catheter ablation	Cohort study	9.7 ± 2.4 months	AF recurrence was defined as a documented AF for more than 30 seconds after three months of a blanking-period.	53±29 months	8
Shim 2013	Korea	575	Patients with AF who underwent radiofrequency catheter ablation.	Cohort study	15 ± 7 months	If any ECG documented an AF episode within the three-month blanking period during follow-up, the patient was diagnosed with an early recurrence, and any AF recurrence thereafter was diagnosed with clinical recurrence.	NA	8
Smit 2012	Netherland	100	Patients were included if they had short-lasting persistent AF, defined as a total AF history of, 2 years, a total persistent AF history of, 6 months, and ≤1 previous electrical cardioversion.	Cohort study	12 months	The primary endpoint consisted of early AF recurrence, defined as any (a)symptomatic recurrence of AF within the first month after cardioversion lasting ≥30s. Secondary endpoint was progression to permanent AF within 1 year.	4.2 months	9
Canpolat 2014	Turkey	41	Lone paroxysmal AF patients who underwent preablation DE-MRI. Lone AF was defined in patients who were <60 years old; without structural heart disease based on patient history, physical examination, and imaging methods including chest X-ray and echocardiography; and no history of coronary artery disease, diabetes mellitus, or hypertension. Paroxysmal AF is defined as self-terminating episode, usually within 48 hours, that may continue for up to 7 days.	Cohort study	18 months	Recurrence of AF is defined as detection of AF (at least 30 seconds duration when assessed with ECG monitoring) >3 months following AF ablation.	60 months	8
Rosenberg 2014	America	1538	Participants were recruited for The Cardiovascular Health Study.	Cohort study	12 months	The occurrence of AF. (1) Annual outpatient study ECGs were interpreted by the EPICARE ECG reading center, where the diagnoses of AF or atrial flutter were verified; (2) hospital discharge diagnoses that included codes for AF and flutter were also included, although AF or flutter diagnoses that were made during the same hospitalization as coronary artery bypass surgery or heart valve surgery were not counted.	NA	9

AF = atrial fibrillation; RHD = rheumatic heart disease; VHD = valvular heart disease; CHF = congestive heart failure.

**Table 2:** Patients characteristics of included studies.

Investigator (year)	Mean age (y)	Male (%)	BMI (kg/m^2^)	Smoker (%)	HTN (%)	DM (%)	CAD (%)	Mean LAD (mm)	Mean LVEF (%)	Medication			
										β-blocker (%)	ACEI/ARB (%)	Statin (%)	Amiodarone (%)
Wang 2010	45.9	-	25.8	56.1	100	0	0	-	-	0	0	0	0,
Wu 2013	52.8	91.3	26.2	-	28.3	2.2	2.2	44.6	54.6	-	23.9	4.3	26.1
On 2009	53.9	44.7	-	23.7	22.4	14.5	-	60.2	-	-	-	-	-
Xiao 2010	41.2 ± 9.1	-	-	-	0	-	0	50.6	52.9	-	0	-	-
Zhao 2014	50.8	45	-	-	-	0	-	50.9	54.5	65	58	-	-
Kim 2009	58.6	77	24.7	-	28.4	-	-	45.8	49.1	24.3	35.1	10.8	64.9
Mira 2013	49.5	57.5	-	-	-	-	-	57.3	60.5	-	0	-	-
Lin 2015	67.5	62.5	-	32.1	100	21.4	-	38.5	61.6	0	0	0	0
Kimura 2014	59 ± 8	-	23.2 ± 2.6	-	-	-	-	39 ± 6	71.9 ± 9.4	37.9	20.7	17.2	-
Shim 2013	55.7 ± 10.9	77.7	24.8 ± 2.8	-	44	10.3	-	41.4 ± 6.2	61.3 ± 8.3	0	0	0	0
Smit 2012	65 ± 9	74.	-	62	67	14	18	45 ± 6	19 ± 13	89	74	38	12
Canpolat 2014	49.2 ± 7.6	58.5	27.1±5.2	31.7	0	0	0	41.2	68.2±4.5	-	-	-	31.7
Rosenberg 2014	77.8 ± 4.6	37.9	-	9.2	56.3	16.8	-	-	-	-	-	-	-

BMI = body mass index; HNT = hypertension; DM = diabetes mellitus; CAD = coronary artery disease; LAD = left atrium diameter; LVEF = left ventricular ejectionfraction; ACEI = angiotensin converting enzyme inhibitors; ARB = angiotensin receptor blocker.

**Table 3:** Confounding factors used in multivariate analysis.

Investigator (year)	HR/OR	95%CI	P value	Adjustment
Canpolat 2014	HR:1.013(Univariate model)	1.010–1.018	0.001	NA
Smit 2012	HR:1.2	1.0–1.5	0.04	Left ventricular ejection fraction, and early AF recurrence.
Wu 2013	OR:1.11	1.01–1.22	0.031	Age, sex, body mass index, use of angiotensin-converting enzyme inhibitor/ angiotensin II receptor blocker, left atrial diameter.
Rosenberg 2014	HR:1.05	0.95–1.17	0.36	Age, sex, race, clinic site, systolic blood pressure, hypertensive medications, body mass index, body mass index -squared, height, smoking status, history of CHF, MI, or prevalent diabetes.

HR = hazard ratio; OR, odds ratio; CI = confidence interval; NA = not available; AF = atrial fibrillation; CHF = congestive heart failure; MI = myocardial infarction.

### Main analysis

**Fig 2:**
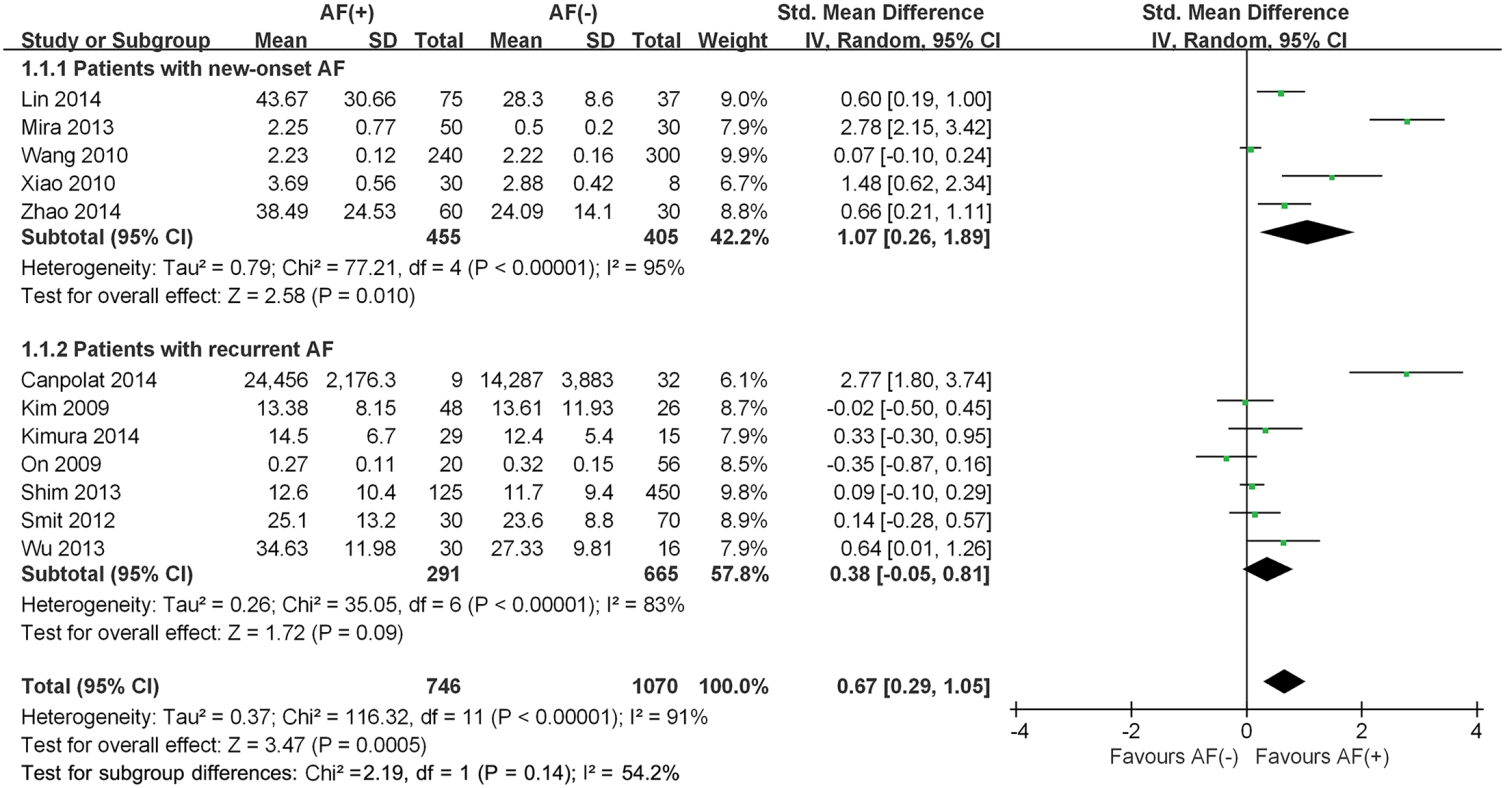
Forest plot of the association between the plasma level of TGF-β1 and AF occurrence depending on different study population in which TGF-β1 levels were analyzed as continuous variable. AF, atrial fibrillation; CI, confidence interval; SD, standard deviation.

**Fig 3:**
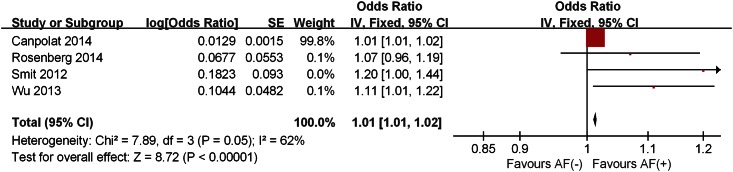
Forest plot of the association between the plasma level of TGF-β1 and AF occurrence in which TGF-β1 levels were analyzed as a categorical variable. AF, atrial fibrillation; CI, confidence interval; OR, odds ratio.

**Fig 4:**
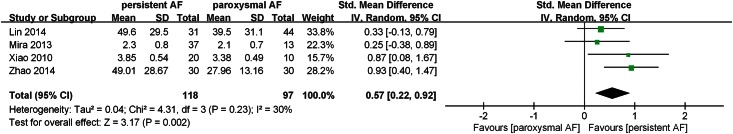
Forest plot of the association between the plasma level of TGF-β1 and the two different types of AF. AF, atrial fibrillation; CI, confidence interval; SD, standard deviation.

The pooled analysis of included studies showed that plasma TGF-β1 levels in the patients with AF was significantly higher than those without AF in both analyses; continuous variable (SMD 0.67; 95%CI 0.29–1.05) with significant heterogeneity across studies (I² = 91%, P<0.00001) (Fig 2) and categorical variable (OR 1.01, 95% CI 1.01–1.02) with moderate heterogeneity across studies (I² = 62%, P = 0.05) (Fig 3). Patients with persistent AF had higher TGF-β1 levels than that in paroxysmal AF patients (SMD 0.57; 95%CI 0.22–0.92) without significant heterogeneity (I² = 30%, P = 0.23) across studies (Fig 4).

### Sensitivity and subgroup analysis

A subgroup analysis was performed based on the type of AF on 5 studies and it showed a positive correlation between high TGF-β1 plasma levels and the risk of new-onset AF (SMD 1.07; 95%CI 0.26–1.89) with significant heterogeneity (I² = 95%, P<0.00001) across studies. However, there was no clear relationship between plasma TGF-β1 levels and the risk of recurrent AF (SMD 0.38; 95%CI (-0.05–0.81) with significant heterogeneity (I² = 83%, P<0.00001) across studies (Fig 2).

A predefined subgroup analysis was performed to investigate the origin of the heterogeneity between studies. In the subgroups with follow-up <12 months, LVEF <50% and sample size ≥100, there were no significant heterogeneity between studies. Therefore, the follow-up duration, LVEF and sample size are likely the origin of the significant heterogeneity in our meta-analysis (Table 4).

**Table 4:** Subgroup analyses of the association between the TGF-β plasma levels and incidence of AF.

Subgroup	Study	Number of studies	Heterogeneity		Meta-analysis		
			I^2^	p-Value	SMD	95% CI	p-Value
Follow-up duration	<12 months	2	0%	0.49	0.48	[0.04, 0.92]	0.03
	≥12 months	5	88%	<0.00001	0.36	[-0.19, 0.92]	0.2
Study design	Cohort	7	83%	<0.00001	0.38	[-0.05, 0.81]	0.09
	Case control	5	95%	<0.00001	1.07	[0.26, 1.89]	0.01
LVEF	<50%	2	0%	0.61	0.07	[-0.25, 0.39]	0.67
	≥50%	8	92	<0.00001	0.81	[0.36, 1.26]	0.0004
Sample size	<100	8	92%	<0.00001	1	[0.24, 1.75]	0.01
	≥100	4	48%	0.12	0.17	[-0.02, 0.35]	0.07
Age of patients	≤50 years	4	97%	<0.00001	1.75	[0.09, 3.41]	0.04
	>50 years	8	57%	0.02	0.25	[0.01, 0.48]	0.04

AF = atrial fibrillation; LVEF = left ventricular ejection fraction; CI = confidence interval; SMD = standard mean difference.

**Fig 5:**
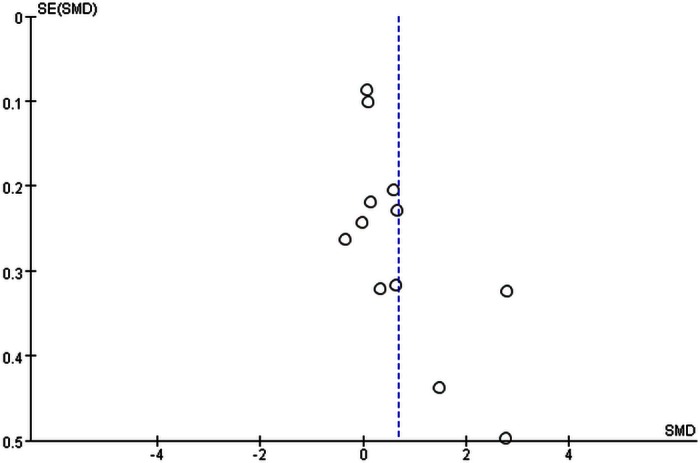
Funnel plot of the meta-analysis.

**Fig 6:**
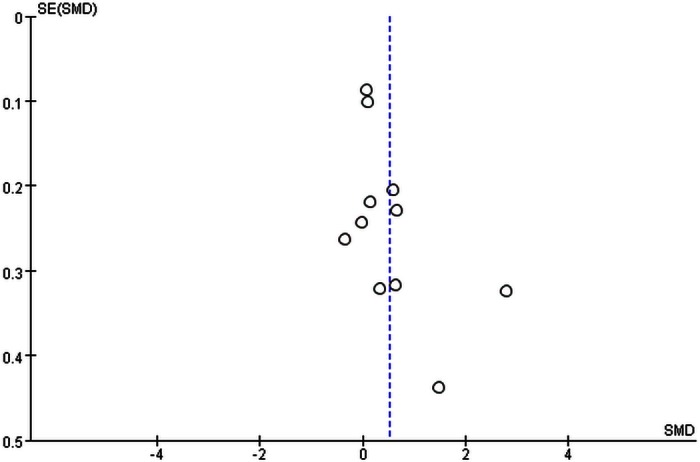
Funnel plot after removing the study with the highest levels of TGF-β1.

Finally, we performed a sensitivity analysis and found that there was no significant difference on the overall heterogeneity regardless of which study was removed. The result of the funnel plot for TGF-β1 in AF patients was asymmetrical, indicating the potential for publication bias (Fig 5). After removing the study with the highest levels of TGF-β1, the result of the funnel plot was symmetrical (Fig 6).

## Discussion

The main finding of this comprehensive meta-analysis is that there is an association between high plasma TGF-β1 levels and risk of AF, especially for new-onset AF. To the best of our knowledge, this is the first comprehensive meta-analysis performed to investigate their relationship.

TGF-β1 is a major factor promoting collagen production in cardiac fibroblasts [27]. It is also considered to be a key factor in the signal cascade reaction during the process of tissue fibrosis [28, 29]. Multiple studies have found increased atrial fibrosis on biopsy or autopsy specimens of patients with AF and concurrently elevated plasma level of TGF-β1 [30, 31, 32]. Previous studies have only investigated the plasma level of TGF-β1 in specific subgroups, such as the patients who developed recurrent AF (following surgical maze procedure, electrical cardioversion and catheter ablation) or patients with new-onset AF after cardiac surgery. In our meta-analysis, different groups were analyzed together to identify the potential role of TGF-β1 in promoting AF. We demonstrated that there is a positive correlation between higher plasma TGF-β1 levels and the development of new onset AF and the overall occurrence of AF. However, it is worth noting that there was no clear relationship between plasma TGF-β1 levels and recurrent AF in the subgroup analysis. This finding could be due to the heterogeneity in study population and AF management strategy between studies.

4 studies included information on the type of AF, whether they were persistent or paroxysmal AF. Persistent AF was defined as AF lasting for more than 7 days while paroxysmal AF was defined as AF with spontaneous termination in less than 7 days. To investigate the relationship between TGF-β1 and the type of AF, we analyzed these 4[14, 16–18] studies which included the plasma level of TGF-β1 in both persistent AF and paroxysmal AF patients. TGF-β1 levels were found to be higher in patients with persistent AF compared to paroxysmal AF. The finding was expected as atrial fibrosis is more extensive with longer duration of AF. It also leads us to the hypothesis that TGF-β1 as an index of atrial fibrosis may inform us of the chronicity of AF.

Despite being the most common sustained arrhythmia, the mechanism of AF is poorly understood. Systemic inflammatory response appear to be the contributing factor to the occurrence and recurrence of AF. A meta-analysis performed by Wu et al [33] demonstrated that high levels of circulating inflammatory factors especially CRP and IL-6 are associated with greater risk of AF in the general population, occurrence of AF after coronary artery bypass grafting and AF recurrence after electrical cardioversion or catheter ablation. In our analysis, we found that elevated levels of plasma TGF-β1 was associated with the occurrence of new onset AF. Our findings provide important insight into the mechanisms of AF.

## Study Limitations

The present meta-analysis has several limitations. First, AF duration and methods of AF detection were different among the studies which account for the heterogeneity between the individual studies. Second, the sample size of the meta-analysis was relatively small. Finally, the asymmetrical funnel plot suggests that there may be publication bias.

## Conclusions

In conclusion, this meta-analysis suggests an association between high plasma TGF-β1 and the occurrence of new onset AF. Additional studies with larger sample sizes are needed to further investigate the relationship between plasma TGF-β1 and the occurrence of AF.

## Supporting Information

S1 File PRISMA checklist. (DOC)

S2 File Exclusion reasons for 26 articles. (DOC)
