# Supplementary material for: Association of Plasma Transforming Growth Factor-β1 Levels and the Risk of Atrial Fibrillation: A Meta-Analysis
Source: PLoS One. 2016 May 12;11(5):e0155275. doi: 10.1371/journal.pone.0155275 (PMC4865111; doi:10.1371/journal.pone.0155275)
Supplement: S2 File — (DOC) [file pone.0155275.s002.doc]

**Excluding reasons of 26 full-text excluded articles:**

**17 didn’t provide the plasma levels of TGF-β1:**

# 1. [Yi SL](http://www.ncbi.nlm.nih.gov/pubmed/?term=Yi SL%5BAuthor%5D&cauthor=true&cauthor_uid=24454806), [Liu XJ](http://www.ncbi.nlm.nih.gov/pubmed/?term=Liu XJ%5BAuthor%5D&cauthor=true&cauthor_uid=24454806), [Zhong JQ](http://www.ncbi.nlm.nih.gov/pubmed/?term=Zhong JQ%5BAuthor%5D&cauthor=true&cauthor_uid=24454806), [Zhang Y](http://www.ncbi.nlm.nih.gov/pubmed/?term=Zhang Y%5BAuthor%5D&cauthor=true&cauthor_uid=24454806): Role of caveolin-1 in atrial fibrillation as an anti-fibrotic signaling molecular in human atrial fibroblasts. [PLoS One.](http://www.ncbi.nlm.nih.gov/pubmed/?term=Role+of+Caveolin-1+in+Atrial+Fibrillation+as+an+Anti-Fibrotic+Signaling+Molecule+in+Human+Atrial+Fibroblasts) 2014 Jan 14;9(1).

2. [Zhang D](http://www.ncbi.nlm.nih.gov/pubmed/?term=Zhang D%5BAuthor%5D&cauthor=true&cauthor_uid=25376239), [Liu X](http://www.ncbi.nlm.nih.gov/pubmed/?term=Liu X%5BAuthor%5D&cauthor=true&cauthor_uid=25376239), [Chen X](http://www.ncbi.nlm.nih.gov/pubmed/?term=Chen X%5BAuthor%5D&cauthor=true&cauthor_uid=25376239), [Gu J](http://www.ncbi.nlm.nih.gov/pubmed/?term=Gu J%5BAuthor%5D&cauthor=true&cauthor_uid=25376239), [Li F](http://www.ncbi.nlm.nih.gov/pubmed/?term=Li F%5BAuthor%5D&cauthor=true&cauthor_uid=25376239), [Zhang W](http://www.ncbi.nlm.nih.gov/pubmed/?term=Zhang W%5BAuthor%5D&cauthor=true&cauthor_uid=25376239), [Zheng Y](http://www.ncbi.nlm.nih.gov/pubmed/?term=Zheng Y%5BAuthor%5D&cauthor=true&cauthor_uid=25376239): Role of the MAPKs/TGF-β1/TRAF6 Signaling Pathway in Atrial Fibrosis of Patients with Chronic Atrial Fibrillation and Rheumatic Mitral Valve Disease. [Cardiology.](http://www.ncbi.nlm.nih.gov/pubmed/?term=Role+of+the+MAPKs%2FTGF-β1%2FTRAF6+Signaling+Pathway+in+Atrial+Fibrosis+of+Patients+with+Chronic+Atrial+Fibrillation+and+Rheumatic+Mitral+Valve+Disease) 2014 129(4):216-23.

3. [Cao H](http://www.ncbi.nlm.nih.gov/pubmed/?term=Cao H%5BAuthor%5D&cauthor=true&cauthor_uid=25402477), [Zhou Q](http://www.ncbi.nlm.nih.gov/pubmed/?term=Zhou Q%5BAuthor%5D&cauthor=true&cauthor_uid=25402477), [Lan R](http://www.ncbi.nlm.nih.gov/pubmed/?term=Lan R%5BAuthor%5D&cauthor=true&cauthor_uid=25402477), [Røe OD](http://www.ncbi.nlm.nih.gov/pubmed/?term=Røe OD%5BAuthor%5D&cauthor=true&cauthor_uid=25402477), [Chen X](http://www.ncbi.nlm.nih.gov/pubmed/?term=Chen X%5BAuthor%5D&cauthor=true&cauthor_uid=25402477), [Chen Y](http://www.ncbi.nlm.nih.gov/pubmed/?term=Chen Y%5BAuthor%5D&cauthor=true&cauthor_uid=25402477), [Wang D](http://www.ncbi.nlm.nih.gov/pubmed/?term=Wang D%5BAuthor%5D&cauthor=true&cauthor_uid=25402477): A Functional Polymorphism C-509T in TGFb-1 Promoter Contributes to Susceptibility and Prognosis of Lone Atrial Fibrillation in Chinese Population. [PLoS One.](http://www.ncbi.nlm.nih.gov/pubmed/?term=A+Functional+Polymorphism+C-509T+in+TGFb-1+Promoter+Contributes+to+Susceptibility+and+Prognosis+of+Lone+Atrial+Fibrillation+in+Chinese+Population) 2014 Nov 17;9(11).

4. [Aldhoon B](http://www.ncbi.nlm.nih.gov/pubmed/?term=Aldhoon B%5BAuthor%5D&cauthor=true&cauthor_uid=23489193), [Kučera T](http://www.ncbi.nlm.nih.gov/pubmed/?term=Kučera T%5BAuthor%5D&cauthor=true&cauthor_uid=23489193), [Smorodinová N](http://www.ncbi.nlm.nih.gov/pubmed/?term=Smorodinová N%5BAuthor%5D&cauthor=true&cauthor_uid=23489193), [Martínek J](http://www.ncbi.nlm.nih.gov/pubmed/?term=Martínek J%5BAuthor%5D&cauthor=true&cauthor_uid=23489193), [Melenovský V](http://www.ncbi.nlm.nih.gov/pubmed/?term=Melenovský V%5BAuthor%5D&cauthor=true&cauthor_uid=23489193), [Kautzner J](http://www.ncbi.nlm.nih.gov/pubmed/?term=Kautzner J%5BAuthor%5D&cauthor=true&cauthor_uid=23489193): Associations Between Cardiac Fibrosis and Permanent Atrial Fibrillation in Advanced Heart Failure. [Physiol Res.](http://www.ncbi.nlm.nih.gov/pubmed/?term=Associations+Between+Cardiac+Fibrosis+and+Permanent+Atrial+Fibrillation+in+Advanced+Heart+Failure) 2013;62(3):247-55.

5. [Polyakova V](http://www.ncbi.nlm.nih.gov/pubmed/?term=Polyakova V%5BAuthor%5D&cauthor=true&cauthor_uid=18194448), [Miyagawa S](http://www.ncbi.nlm.nih.gov/pubmed/?term=Miyagawa S%5BAuthor%5D&cauthor=true&cauthor_uid=18194448), [Szalay Z](http://www.ncbi.nlm.nih.gov/pubmed/?term=Szalay Z%5BAuthor%5D&cauthor=true&cauthor_uid=18194448), [Risteli J](http://www.ncbi.nlm.nih.gov/pubmed/?term=Risteli J%5BAuthor%5D&cauthor=true&cauthor_uid=18194448), [Kostin S](http://www.ncbi.nlm.nih.gov/pubmed/?term=Kostin S%5BAuthor%5D&cauthor=true&cauthor_uid=18194448): Atrial extracellular matrix remodelling in patients with atrial fibrillation. [J Cell Mol Med.](http://www.ncbi.nlm.nih.gov/pubmed/18194448) 2008 Jan-Feb;12(1):189-208.

6. [Gramley F](http://www.ncbi.nlm.nih.gov/pubmed/?term=Gramley F%5BAuthor%5D&cauthor=true&cauthor_uid=19394095), [Lorenzen J](http://www.ncbi.nlm.nih.gov/pubmed/?term=Lorenzen J%5BAuthor%5D&cauthor=true&cauthor_uid=19394095), [Koellensperger E](http://www.ncbi.nlm.nih.gov/pubmed/?term=Koellensperger E%5BAuthor%5D&cauthor=true&cauthor_uid=19394095), [Kettering K](http://www.ncbi.nlm.nih.gov/pubmed/?term=Kettering K%5BAuthor%5D&cauthor=true&cauthor_uid=19394095), [Weiss C](http://www.ncbi.nlm.nih.gov/pubmed/?term=Weiss C%5BAuthor%5D&cauthor=true&cauthor_uid=19394095), [Munzel T](http://www.ncbi.nlm.nih.gov/pubmed/?term=Munzel T%5BAuthor%5D&cauthor=true&cauthor_uid=19394095): Atrial fibrosis and atrial fibrillation: The role of the TGF-β1 signaling pathway. [Int J Cardiol.](http://www.ncbi.nlm.nih.gov/pubmed/19394095) 2010 Sep 3;143(3):405-13.

7. [Wang W](http://www.ncbi.nlm.nih.gov/pubmed/?term=Wang W%5BAuthor%5D&cauthor=true&cauthor_uid=22412222), [Liu L](http://www.ncbi.nlm.nih.gov/pubmed/?term=Liu L%5BAuthor%5D&cauthor=true&cauthor_uid=22412222), [Li Y](http://www.ncbi.nlm.nih.gov/pubmed/?term=Li Y%5BAuthor%5D&cauthor=true&cauthor_uid=22412222), [Hu SS](http://www.ncbi.nlm.nih.gov/pubmed/?term=Hu SS%5BAuthor%5D&cauthor=true&cauthor_uid=22412222), [Song YH](http://www.ncbi.nlm.nih.gov/pubmed/?term=Song YH%5BAuthor%5D&cauthor=true&cauthor_uid=22412222), [Wang X](http://www.ncbi.nlm.nih.gov/pubmed/?term=Wang X%5BAuthor%5D&cauthor=true&cauthor_uid=22412222): Does the Expression of Transforming Growth Factor β-1 Affect the Outcome of the Radiofrequency Modified Maze Procedure in Patients with Rheumatic Atrial Fibrillation? [Tex Heart Inst J.](http://www.ncbi.nlm.nih.gov/pubmed/?term=Does+the+Expression+of+Transforming+Growth+Factor+β-1+Affect+the+Outcome+of+the+Radiofrequency+Modified+Maze+Procedure+in+Patients+with+Rheumatic+Atrial+Fibrillation%3F) 2012;39(1):17-23.

8. [Swartz MF](http://www.ncbi.nlm.nih.gov/pubmed/?term=Swartz MF%5BAuthor%5D&cauthor=true&cauthor_uid=23040566), [Fink GW](http://www.ncbi.nlm.nih.gov/pubmed/?term=Fink GW%5BAuthor%5D&cauthor=true&cauthor_uid=23040566), [Sarwar MF](http://www.ncbi.nlm.nih.gov/pubmed/?term=Sarwar MF%5BAuthor%5D&cauthor=true&cauthor_uid=23040566), [Hicks GL](http://www.ncbi.nlm.nih.gov/pubmed/?term=Hicks GL%5BAuthor%5D&cauthor=true&cauthor_uid=23040566), [Yu Y](http://www.ncbi.nlm.nih.gov/pubmed/?term=Yu Y%5BAuthor%5D&cauthor=true&cauthor_uid=23040566), [Hu R](http://www.ncbi.nlm.nih.gov/pubmed/?term=Hu R%5BAuthor%5D&cauthor=true&cauthor_uid=23040566), [Lutz CJ](http://www.ncbi.nlm.nih.gov/pubmed/?term=Lutz CJ%5BAuthor%5D&cauthor=true&cauthor_uid=23040566), [Taffet SM](http://www.ncbi.nlm.nih.gov/pubmed/?term=Taffet SM%5BAuthor%5D&cauthor=true&cauthor_uid=23040566), [Jalife J](http://www.ncbi.nlm.nih.gov/pubmed/?term=Jalife J%5BAuthor%5D&cauthor=true&cauthor_uid=23040566): Elevated Pre-Operative Serum Peptides for Collagen I and III Synthesis Result in Post-Surgical Atrial Fibrillation. [J Am Coll Cardiol.](http://www.ncbi.nlm.nih.gov/pubmed/?term=Elevated+Pre-Operative+Serum+Peptides+for+Collagen+I+and+III+Synthesis+Result+in+Post-Surgical+Atrial+Fibrillation) 2012 Oct 30;60(18):1799-806.

9. [Zhang P](http://www.ncbi.nlm.nih.gov/pubmed/?term=Zhang P%5BAuthor%5D&cauthor=true&cauthor_uid=23639457), [Wang W](http://www.ncbi.nlm.nih.gov/pubmed/?term=Wang W%5BAuthor%5D&cauthor=true&cauthor_uid=23639457), [Wang X](http://www.ncbi.nlm.nih.gov/pubmed/?term=Wang X%5BAuthor%5D&cauthor=true&cauthor_uid=23639457), [Wang X](http://www.ncbi.nlm.nih.gov/pubmed/?term=Wang X%5BAuthor%5D&cauthor=true&cauthor_uid=23639457), [Song Y](http://www.ncbi.nlm.nih.gov/pubmed/?term=Song Y%5BAuthor%5D&cauthor=true&cauthor_uid=23639457), [Zhang J](http://www.ncbi.nlm.nih.gov/pubmed/?term=Zhang J%5BAuthor%5D&cauthor=true&cauthor_uid=23639457), [Zhao H](http://www.ncbi.nlm.nih.gov/pubmed/?term=Zhao H%5BAuthor%5D&cauthor=true&cauthor_uid=23639457): Focal adhesion kinase mediates atrial fibrosis via the AKT/S6K signaling pathway in chronic atrial fibrillation patients with rheumatic mitral valve disease. [Int J Cardiol.](http://www.ncbi.nlm.nih.gov/pubmed/?term=Focal+adhesion+kinase+mediates+atrial+fibrosis+via+the+AKT%2FS6K+signaling+pathway+in+chronic+atrial+fibrillation+patients+with+rheumatic+mitral+valve+disease) 2013 Oct 9;168(4):3200-7.

10. [Zhou J](http://www.ncbi.nlm.nih.gov/pubmed/?term=Zhou J%5BAuthor%5D&cauthor=true&cauthor_uid=24463922), [Gao J](http://www.ncbi.nlm.nih.gov/pubmed/?term=Gao J%5BAuthor%5D&cauthor=true&cauthor_uid=24463922), [Liu Y](http://www.ncbi.nlm.nih.gov/pubmed/?term=Liu Y%5BAuthor%5D&cauthor=true&cauthor_uid=24463922), [Gu S](http://www.ncbi.nlm.nih.gov/pubmed/?term=Gu S%5BAuthor%5D&cauthor=true&cauthor_uid=24463922), [Zhang X](http://www.ncbi.nlm.nih.gov/pubmed/?term=Zhang X%5BAuthor%5D&cauthor=true&cauthor_uid=24463922), [An X](http://www.ncbi.nlm.nih.gov/pubmed/?term=An X%5BAuthor%5D&cauthor=true&cauthor_uid=24463922), [Yan J](http://www.ncbi.nlm.nih.gov/pubmed/?term=Yan J%5BAuthor%5D&cauthor=true&cauthor_uid=24463922), [Xin Y](http://www.ncbi.nlm.nih.gov/pubmed/?term=Xin Y%5BAuthor%5D&cauthor=true&cauthor_uid=24463922), [Su P](http://www.ncbi.nlm.nih.gov/pubmed/?term=Su P%5BAuthor%5D&cauthor=true&cauthor_uid=24463922): Human Atrium Transcript Analysis of Permanent Atrial Fibrillation. [Int Heart J.](http://www.ncbi.nlm.nih.gov/pubmed/?term=Human+Atrium+Transcript+Analysis+of+Permanent+Atrial+Fibrillation) 2014;55(1):71-7.

11. [Adam O](http://www.ncbi.nlm.nih.gov/pubmed/?term=Adam O%5BAuthor%5D&cauthor=true&cauthor_uid=20117462), [Lavall D](http://www.ncbi.nlm.nih.gov/pubmed/?term=Lavall D%5BAuthor%5D&cauthor=true&cauthor_uid=20117462), [Theobald K](http://www.ncbi.nlm.nih.gov/pubmed/?term=Theobald K%5BAuthor%5D&cauthor=true&cauthor_uid=20117462), [Hohl M](http://www.ncbi.nlm.nih.gov/pubmed/?term=Hohl M%5BAuthor%5D&cauthor=true&cauthor_uid=20117462), [Grube M](http://www.ncbi.nlm.nih.gov/pubmed/?term=Grube M%5BAuthor%5D&cauthor=true&cauthor_uid=20117462), [Ameling S](http://www.ncbi.nlm.nih.gov/pubmed/?term=Ameling S%5BAuthor%5D&cauthor=true&cauthor_uid=20117462), [Sussman MA](http://www.ncbi.nlm.nih.gov/pubmed/?term=Sussman MA%5BAuthor%5D&cauthor=true&cauthor_uid=20117462), [Rosenkranz S](http://www.ncbi.nlm.nih.gov/pubmed/?term=Rosenkranz S%5BAuthor%5D&cauthor=true&cauthor_uid=20117462), [Kroemer HK](http://www.ncbi.nlm.nih.gov/pubmed/?term=Kroemer HK%5BAuthor%5D&cauthor=true&cauthor_uid=20117462), [Schäfers HJ](http://www.ncbi.nlm.nih.gov/pubmed/?term=Schäfers HJ%5BAuthor%5D&cauthor=true&cauthor_uid=20117462),[Böhm M](http://www.ncbi.nlm.nih.gov/pubmed/?term=Böhm M%5BAuthor%5D&cauthor=true&cauthor_uid=20117462), [Laufs U](http://www.ncbi.nlm.nih.gov/pubmed/?term=Laufs U%5BAuthor%5D&cauthor=true&cauthor_uid=20117462): Rac1-Induced Connective Tissue Growth Factor Regulates Connexin 43 and N-Cadherin Expression in Atrial Fibrillation. [J Am Coll Cardiol.](http://www.ncbi.nlm.nih.gov/pubmed/?term=Rac1-Induced+Connective+Tissue+Growth+Factor+Regulates+Connexin+43+and+N-Cadherin+Expression+in+Atrial+Fibrillation) 2010 Feb 2;55(5):469-80.

12. [Yamashita T](http://www.ncbi.nlm.nih.gov/pubmed/?term=Yamashita T%5BAuthor%5D&cauthor=true&cauthor_uid=20009387), [Sekiguchi A](http://www.ncbi.nlm.nih.gov/pubmed/?term=Sekiguchi A%5BAuthor%5D&cauthor=true&cauthor_uid=20009387), [Iwasaki YK](http://www.ncbi.nlm.nih.gov/pubmed/?term=Iwasaki YK%5BAuthor%5D&cauthor=true&cauthor_uid=20009387), [Date T](http://www.ncbi.nlm.nih.gov/pubmed/?term=Date T%5BAuthor%5D&cauthor=true&cauthor_uid=20009387), [Sagara K](http://www.ncbi.nlm.nih.gov/pubmed/?term=Sagara K%5BAuthor%5D&cauthor=true&cauthor_uid=20009387), [Tanabe H](http://www.ncbi.nlm.nih.gov/pubmed/?term=Tanabe H%5BAuthor%5D&cauthor=true&cauthor_uid=20009387), [Suma H](http://www.ncbi.nlm.nih.gov/pubmed/?term=Suma H%5BAuthor%5D&cauthor=true&cauthor_uid=20009387), [Sawada H](http://www.ncbi.nlm.nih.gov/pubmed/?term=Sawada H%5BAuthor%5D&cauthor=true&cauthor_uid=20009387), [Aizawa T](http://www.ncbi.nlm.nih.gov/pubmed/?term=Aizawa T%5BAuthor%5D&cauthor=true&cauthor_uid=20009387): Recruitment of Immune Cells Across Atrial Endocardium in Human Atrial Fibrillation. [Circ J.](http://www.ncbi.nlm.nih.gov/pubmed/?term=Recruitment+of+Immune+Cells+Across+Atrial+Endocardium+in+Human+Atrial+Fibrillation) 2010 Feb;74(2):262-70.

13. [Goette A](http://www.ncbi.nlm.nih.gov/pubmed/?term=Goette A%5BAuthor%5D&cauthor=true&cauthor_uid=12063299), [Arndt M](http://www.ncbi.nlm.nih.gov/pubmed/?term=Arndt M%5BAuthor%5D&cauthor=true&cauthor_uid=12063299), [Röcken C](http://www.ncbi.nlm.nih.gov/pubmed/?term=Röcken C%5BAuthor%5D&cauthor=true&cauthor_uid=12063299), [Staack T](http://www.ncbi.nlm.nih.gov/pubmed/?term=Staack T%5BAuthor%5D&cauthor=true&cauthor_uid=12063299), [Bechtloff R](http://www.ncbi.nlm.nih.gov/pubmed/?term=Bechtloff R%5BAuthor%5D&cauthor=true&cauthor_uid=12063299), [Reinhold D](http://www.ncbi.nlm.nih.gov/pubmed/?term=Reinhold D%5BAuthor%5D&cauthor=true&cauthor_uid=12063299), [Huth C](http://www.ncbi.nlm.nih.gov/pubmed/?term=Huth C%5BAuthor%5D&cauthor=true&cauthor_uid=12063299), [Ansorge S](http://www.ncbi.nlm.nih.gov/pubmed/?term=Ansorge S%5BAuthor%5D&cauthor=true&cauthor_uid=12063299), [Klein HU](http://www.ncbi.nlm.nih.gov/pubmed/?term=Klein HU%5BAuthor%5D&cauthor=true&cauthor_uid=12063299), [Lendeckel U](http://www.ncbi.nlm.nih.gov/pubmed/?term=Lendeckel U%5BAuthor%5D&cauthor=true&cauthor_uid=12063299): Calpains and cytokines in fibrillating human atria. [Am J Physiol Heart Circ Physiol.](http://www.ncbi.nlm.nih.gov/pubmed/?term=Calpains+and+cytokines+in+fibrillating+human+atria) 2002 Jul;283(1):H264-72.

14. [Zhao F](http://www.ncbi.nlm.nih.gov/pubmed/?term=Zhao F%5BAuthor%5D&cauthor=true&cauthor_uid=23465222), [Zhang S](http://www.ncbi.nlm.nih.gov/pubmed/?term=Zhang S%5BAuthor%5D&cauthor=true&cauthor_uid=23465222), [Shao Y](http://www.ncbi.nlm.nih.gov/pubmed/?term=Shao Y%5BAuthor%5D&cauthor=true&cauthor_uid=23465222), [Wu Y](http://www.ncbi.nlm.nih.gov/pubmed/?term=Wu Y%5BAuthor%5D&cauthor=true&cauthor_uid=23465222), [Qin J](http://www.ncbi.nlm.nih.gov/pubmed/?term=Qin J%5BAuthor%5D&cauthor=true&cauthor_uid=23465222), [Chen Y](http://www.ncbi.nlm.nih.gov/pubmed/?term=Chen Y%5BAuthor%5D&cauthor=true&cauthor_uid=23465222), [Chen L](http://www.ncbi.nlm.nih.gov/pubmed/?term=Chen L%5BAuthor%5D&cauthor=true&cauthor_uid=23465222), [Gu H](http://www.ncbi.nlm.nih.gov/pubmed/?term=Gu H%5BAuthor%5D&cauthor=true&cauthor_uid=23465222), [Wang X](http://www.ncbi.nlm.nih.gov/pubmed/?term=Wang X%5BAuthor%5D&cauthor=true&cauthor_uid=23465222), [Huang C](http://www.ncbi.nlm.nih.gov/pubmed/?term=Huang C%5BAuthor%5D&cauthor=true&cauthor_uid=23465222), [Zhang W](http://www.ncbi.nlm.nih.gov/pubmed/?term=Zhang W%5BAuthor%5D&cauthor=true&cauthor_uid=23465222): Calreticulin overexpression correlates with integrin-α5 and transforming growth factor-β1 expression in the atria of patients with rheumatic valvular disease and atrial fibrillation. [Int J Cardiol.](http://www.ncbi.nlm.nih.gov/pubmed/?term=Calreticulin+overexpression+correlates+with+integrin-α5+and+transforming+growth+factor-β1+expression+in+the+atria+of+patients+with+rheumatic+valvular+disease+and+atrial+fibrillatio) 2013 Oct 3;168(3):2177-85.

15. [Li Y](http://www.ncbi.nlm.nih.gov/pubmed/?term=Li Y%5BAuthor%5D&cauthor=true&cauthor_uid=23571482), [Jian Z](http://www.ncbi.nlm.nih.gov/pubmed/?term=Jian Z%5BAuthor%5D&cauthor=true&cauthor_uid=23571482), [Yang ZY](http://www.ncbi.nlm.nih.gov/pubmed/?term=Yang ZY%5BAuthor%5D&cauthor=true&cauthor_uid=23571482), [Chen L](http://www.ncbi.nlm.nih.gov/pubmed/?term=Chen L%5BAuthor%5D&cauthor=true&cauthor_uid=23571482), [Wang XF](http://www.ncbi.nlm.nih.gov/pubmed/?term=Wang XF%5BAuthor%5D&cauthor=true&cauthor_uid=23571482), [Ma RY](http://www.ncbi.nlm.nih.gov/pubmed/?term=Ma RY%5BAuthor%5D&cauthor=true&cauthor_uid=23571482), [Xiao YB](http://www.ncbi.nlm.nih.gov/pubmed/?term=Xiao YB%5BAuthor%5D&cauthor=true&cauthor_uid=23571482): Increased Expression of Connective Tissue Growth Factor and Transforming Growth Factor-Beta-1 in Atrial Myocardium of Patients with Chronic Atrial Fibrillation. [Cardiology.](http://www.ncbi.nlm.nih.gov/pubmed/?term=Increased+Expression+of+Connective+Tissue+Growth+Factor+and+Transforming+Growth+Factor-Beta-1+in+Atrial+Myocardium+of+Patients+with+Chronic+Atrial+Fibrillation) 2013;124(4):233-40.

16. [Zhang YJ](http://www.ncbi.nlm.nih.gov/pubmed/?term=Zhang YJ%5BAuthor%5D&cauthor=true&cauthor_uid=25796343), [Ma N](http://www.ncbi.nlm.nih.gov/pubmed/?term=Ma N%5BAuthor%5D&cauthor=true&cauthor_uid=25796343), [Su F](http://www.ncbi.nlm.nih.gov/pubmed/?term=Su F%5BAuthor%5D&cauthor=true&cauthor_uid=25796343), [Liu H](http://www.ncbi.nlm.nih.gov/pubmed/?term=Liu H%5BAuthor%5D&cauthor=true&cauthor_uid=25796343), [Mei J](http://www.ncbi.nlm.nih.gov/pubmed/?term=Mei J%5BAuthor%5D&cauthor=true&cauthor_uid=25796343): Increased TRPM6 expression in atrial fibrillation patients contribute to atrial fibrosis. [Exp Mol Pathol.](http://www.ncbi.nlm.nih.gov/pubmed/?term=Increased+TRPM6+expression+in+atrial+fibrillation+patients+contribute+to+atrial+fibrosis) 2015 Jun;98(3):486-90.

# 17. [Sun Y](http://www.ncbi.nlm.nih.gov/pubmed/?term=Sun Y%5BAuthor%5D&cauthor=true&cauthor_uid=25971370), [Huang ZY](http://www.ncbi.nlm.nih.gov/pubmed/?term=Huang ZY%5BAuthor%5D&cauthor=true&cauthor_uid=25971370), [Wang ZH](http://www.ncbi.nlm.nih.gov/pubmed/?term=Wang ZH%5BAuthor%5D&cauthor=true&cauthor_uid=25971370), [Li CP](http://www.ncbi.nlm.nih.gov/pubmed/?term=Li CP%5BAuthor%5D&cauthor=true&cauthor_uid=25971370), [Meng XL](http://www.ncbi.nlm.nih.gov/pubmed/?term=Meng XL%5BAuthor%5D&cauthor=true&cauthor_uid=25971370), [Zhang YJ](http://www.ncbi.nlm.nih.gov/pubmed/?term=Zhang YJ%5BAuthor%5D&cauthor=true&cauthor_uid=25971370), [Su F](http://www.ncbi.nlm.nih.gov/pubmed/?term=Su F%5BAuthor%5D&cauthor=true&cauthor_uid=25971370), [Ma N](http://www.ncbi.nlm.nih.gov/pubmed/?term=Ma N%5BAuthor%5D&cauthor=true&cauthor_uid=25971370): TGF-β1 and TIMP-4 regulate atrial fibrosis in atrial fibrillation secondary to rheumatic heart disease. [Mol Cell Biochem.](http://www.ncbi.nlm.nih.gov/pubmed/?term=TGF-b1+and+TIMP-4+regulate+atrial+ﬁbrosis+in+atrial+ﬁbrillation) 2015 Aug;406(1-2):131-8.

**6 did not provide (mean ± SD) data of TGF-β1 or OR/HR values**

1. [Kim SK](http://www.ncbi.nlm.nih.gov/pubmed/?term=Kim SK%5BAuthor%5D&cauthor=true&cauthor_uid=21186331), [Park JH](http://www.ncbi.nlm.nih.gov/pubmed/?term=Park JH%5BAuthor%5D&cauthor=true&cauthor_uid=21186331), [Kim JY](http://www.ncbi.nlm.nih.gov/pubmed/?term=Kim JY%5BAuthor%5D&cauthor=true&cauthor_uid=21186331), [Choi JI](http://www.ncbi.nlm.nih.gov/pubmed/?term=Choi JI%5BAuthor%5D&cauthor=true&cauthor_uid=21186331), [Joung B](http://www.ncbi.nlm.nih.gov/pubmed/?term=Joung B%5BAuthor%5D&cauthor=true&cauthor_uid=21186331), [Lee MH](http://www.ncbi.nlm.nih.gov/pubmed/?term=Lee MH%5BAuthor%5D&cauthor=true&cauthor_uid=21186331), [Kim SS](http://www.ncbi.nlm.nih.gov/pubmed/?term=Kim SS%5BAuthor%5D&cauthor=true&cauthor_uid=21186331), [Kim YH](http://www.ncbi.nlm.nih.gov/pubmed/?term=Kim YH%5BAuthor%5D&cauthor=true&cauthor_uid=21186331), [Pak HN](http://www.ncbi.nlm.nih.gov/pubmed/?term=Pak HN%5BAuthor%5D&cauthor=true&cauthor_uid=21186331): High Plasma Concentrations of Transforming Growth actor-β and Tissue Inhibitor of Metalloproteinase-1 Potential Non-Invasive Predictors for Electroanatomical Remodeling of Atrium in Patients With Non-Valvular Atrial Fibrillation. [Circ J.](http://www.ncbi.nlm.nih.gov/pubmed/?term=High+Plasma+Concentrations+of+Transforming+Growth+Factor-β+and+Tissue+Inhibitor+of+Metalloproteinase-1+–+Potential+Non-Invasive+Predictors+for+Electroanatomical+Remodeling+of+Atrium+in+Patients+With+Non-Valvular+Atrial+Fibrillation+–) 2011;75(3):557-64.

2. [Bjorgvinsdottir L](http://www.ncbi.nlm.nih.gov/pubmed/?term=Bjorgvinsdottir L%5BAuthor%5D&cauthor=true&cauthor_uid=23948862), [Arnar DO](http://www.ncbi.nlm.nih.gov/pubmed/?term=Arnar DO%5BAuthor%5D&cauthor=true&cauthor_uid=23948862), [Indridason OS](http://www.ncbi.nlm.nih.gov/pubmed/?term=Indridason OS%5BAuthor%5D&cauthor=true&cauthor_uid=23948862), [Heidarsdottir R](http://www.ncbi.nlm.nih.gov/pubmed/?term=Heidarsdottir R%5BAuthor%5D&cauthor=true&cauthor_uid=23948862), [Skogstrand K](http://www.ncbi.nlm.nih.gov/pubmed/?term=Skogstrand K%5BAuthor%5D&cauthor=true&cauthor_uid=23948862), [Torfason B](http://www.ncbi.nlm.nih.gov/pubmed/?term=Torfason B%5BAuthor%5D&cauthor=true&cauthor_uid=23948862), [Hougaard DM](http://www.ncbi.nlm.nih.gov/pubmed/?term=Hougaard DM%5BAuthor%5D&cauthor=true&cauthor_uid=23948862), [Palsson R](http://www.ncbi.nlm.nih.gov/pubmed/?term=Palsson R%5BAuthor%5D&cauthor=true&cauthor_uid=23948862),[Skuladottir GV](http://www.ncbi.nlm.nih.gov/pubmed/?term=Skuladottir GV%5BAuthor%5D&cauthor=true&cauthor_uid=23948862): Do High Levels of n-3 Polyunsaturated Fatty Acids in Cell Membranes Increase the Risk of Postoperative Atrial Fibrillation? [Cardiology.](http://www.ncbi.nlm.nih.gov/pubmed/?term=Do+High+Levels+of+n-3+Polyunsaturated+Fatty+Acids+in+Cell+Membranes+Increase+the+Risk+of+Postoperative+Atrial+Fibrillation%3F) 2013;126(2):107-14.

3. [Sasaki N](http://www.ncbi.nlm.nih.gov/pubmed/?term=Sasaki N%5BAuthor%5D&cauthor=true&cauthor_uid=24554250), [Okumura Y](http://www.ncbi.nlm.nih.gov/pubmed/?term=Okumura Y%5BAuthor%5D&cauthor=true&cauthor_uid=24554250), [Watanabe I](http://www.ncbi.nlm.nih.gov/pubmed/?term=Watanabe I%5BAuthor%5D&cauthor=true&cauthor_uid=24554250), [Mano H](http://www.ncbi.nlm.nih.gov/pubmed/?term=Mano H%5BAuthor%5D&cauthor=true&cauthor_uid=24554250), [Nagashima K](http://www.ncbi.nlm.nih.gov/pubmed/?term=Nagashima K%5BAuthor%5D&cauthor=true&cauthor_uid=24554250), [Sonoda K](http://www.ncbi.nlm.nih.gov/pubmed/?term=Sonoda K%5BAuthor%5D&cauthor=true&cauthor_uid=24554250), [Kogawa R](http://www.ncbi.nlm.nih.gov/pubmed/?term=Kogawa R%5BAuthor%5D&cauthor=true&cauthor_uid=24554250), [Ohkubo K](http://www.ncbi.nlm.nih.gov/pubmed/?term=Ohkubo K%5BAuthor%5D&cauthor=true&cauthor_uid=24554250), [Nakai T](http://www.ncbi.nlm.nih.gov/pubmed/?term=Nakai T%5BAuthor%5D&cauthor=true&cauthor_uid=24554250), [Hirayama A](http://www.ncbi.nlm.nih.gov/pubmed/?term=Hirayama A%5BAuthor%5D&cauthor=true&cauthor_uid=24554250): Increased levels of inflammatory and extracellular matrix turnover biomarkers persist despite reverse atrial structural remodeling during the first year after atrial fibrillation ablation. [J Interv Card Electrophysiol.](http://www.ncbi.nlm.nih.gov/pubmed/?term=Increased+levels+of+inflammatory+and+extracellular+matrix+turnover+biomarkers+persist+despite+reverse+atrial+structural+remodeling+during+the+first+year+after+atrial+fibrillation+ablation) 2014 Apr;39(3):241-9

4. [Richter B](http://www.ncbi.nlm.nih.gov/pubmed/?term=Richter B%5BAuthor%5D&cauthor=true&cauthor_uid=20692054), [Gwechenberger M](http://www.ncbi.nlm.nih.gov/pubmed/?term=Gwechenberger M%5BAuthor%5D&cauthor=true&cauthor_uid=20692054), [Socas A](http://www.ncbi.nlm.nih.gov/pubmed/?term=Socas A%5BAuthor%5D&cauthor=true&cauthor_uid=20692054), [Zorn G](http://www.ncbi.nlm.nih.gov/pubmed/?term=Zorn G%5BAuthor%5D&cauthor=true&cauthor_uid=20692054), [Albinni S](http://www.ncbi.nlm.nih.gov/pubmed/?term=Albinni S%5BAuthor%5D&cauthor=true&cauthor_uid=20692054), [Marx M](http://www.ncbi.nlm.nih.gov/pubmed/?term=Marx M%5BAuthor%5D&cauthor=true&cauthor_uid=20692054), [Wolf F](http://www.ncbi.nlm.nih.gov/pubmed/?term=Wolf F%5BAuthor%5D&cauthor=true&cauthor_uid=20692054), [Bergler-Klein J](http://www.ncbi.nlm.nih.gov/pubmed/?term=Bergler-Klein J%5BAuthor%5D&cauthor=true&cauthor_uid=20692054), [Loewe C](http://www.ncbi.nlm.nih.gov/pubmed/?term=Loewe C%5BAuthor%5D&cauthor=true&cauthor_uid=20692054), [Bieglmayer C](http://www.ncbi.nlm.nih.gov/pubmed/?term=Bieglmayer C%5BAuthor%5D&cauthor=true&cauthor_uid=20692054), [Binder T](http://www.ncbi.nlm.nih.gov/pubmed/?term=Binder T%5BAuthor%5D&cauthor=true&cauthor_uid=20692054),[Wojta J](http://www.ncbi.nlm.nih.gov/pubmed/?term=Wojta J%5BAuthor%5D&cauthor=true&cauthor_uid=20692054), [Gössinger HD](http://www.ncbi.nlm.nih.gov/pubmed/?term=Gössinger HD%5BAuthor%5D&cauthor=true&cauthor_uid=20692054): Time course of markers of tissue repair after ablation of atrial fibrillation and their relation to left atrial structural changes and clinical ablation outcome. [Int J Cardiol.](http://www.ncbi.nlm.nih.gov/pubmed/?term=Time+course+of+markers+of+tissue+repair+after+ablation+of+atrial+fibrillation+and+their+relation+to+left+atrial+structural+changes+and+clinical+ablation+outcome) 2011 Oct 20;152(2):231-6.

5. [Park SJ](http://www.ncbi.nlm.nih.gov/pubmed/?term=Park SJ%5BAuthor%5D&cauthor=true&cauthor_uid=23434344), [On YK](http://www.ncbi.nlm.nih.gov/pubmed/?term=On YK%5BAuthor%5D&cauthor=true&cauthor_uid=23434344), [Kim JS](http://www.ncbi.nlm.nih.gov/pubmed/?term=Kim JS%5BAuthor%5D&cauthor=true&cauthor_uid=23434344), [Choi JO](http://www.ncbi.nlm.nih.gov/pubmed/?term=Choi JO%5BAuthor%5D&cauthor=true&cauthor_uid=23434344), [Ju ES](http://www.ncbi.nlm.nih.gov/pubmed/?term=Ju ES%5BAuthor%5D&cauthor=true&cauthor_uid=23434344), [Jeong DS](http://www.ncbi.nlm.nih.gov/pubmed/?term=Jeong DS%5BAuthor%5D&cauthor=true&cauthor_uid=23434344), [Park PW](http://www.ncbi.nlm.nih.gov/pubmed/?term=Park PW%5BAuthor%5D&cauthor=true&cauthor_uid=23434344), [Jeon ES](http://www.ncbi.nlm.nih.gov/pubmed/?term=Jeon ES%5BAuthor%5D&cauthor=true&cauthor_uid=23434344): Transforming growth factor β1-mediated atrial fibrotic activity and the recovery of atrial mechanical contraction after surgical maze procedure. [Int J Cardiol.](http://www.ncbi.nlm.nih.gov/pubmed/?term=Transforming+growth+factor+β1-mediated+atrial+fibrotic+activity+and+the+recovery+of+atrial+mechanical+contraction+after+surgical+maze+procedure) 2013 Apr 5;164(2):232-7.

6. [Ki MR](http://www.ncbi.nlm.nih.gov/pubmed/?term=Ki MR%5BAuthor%5D&cauthor=true&cauthor_uid=20034688), [Shin DG](http://www.ncbi.nlm.nih.gov/pubmed/?term=Shin DG%5BAuthor%5D&cauthor=true&cauthor_uid=20034688), [Park JS](http://www.ncbi.nlm.nih.gov/pubmed/?term=Park JS%5BAuthor%5D&cauthor=true&cauthor_uid=20034688), [Hong KS](http://www.ncbi.nlm.nih.gov/pubmed/?term=Hong KS%5BAuthor%5D&cauthor=true&cauthor_uid=20034688), [Hong IH](http://www.ncbi.nlm.nih.gov/pubmed/?term=Hong IH%5BAuthor%5D&cauthor=true&cauthor_uid=20034688), [Park JK](http://www.ncbi.nlm.nih.gov/pubmed/?term=Park JK%5BAuthor%5D&cauthor=true&cauthor_uid=20034688), [Jeong KS](http://www.ncbi.nlm.nih.gov/pubmed/?term=Jeong KS%5BAuthor%5D&cauthor=true&cauthor_uid=20034688): Frequency of vacuolating cytotoxin A (VacA)-positive Helicobacter pylori seropositivity and TGF-β1 decrease in atrial fibrillation. [Int J Cardiol.](http://www.ncbi.nlm.nih.gov/pubmed/?term=Frequency+of+vacuolating+cytotoxin+A+(VacA)-positive+Helicobacter+pylori+seropositivity+and+TGF-β1+decrease+in+atrial+fibrillation) 2010 Nov 19;145(2):345-6.

**1 didn’t provide baseline characteristics of patients**

[Seko Y](http://www.ncbi.nlm.nih.gov/pubmed/?term=Seko Y%5BAuthor%5D&cauthor=true&cauthor_uid=10807526), [Nishimura H](http://www.ncbi.nlm.nih.gov/pubmed/?term=Nishimura H%5BAuthor%5D&cauthor=true&cauthor_uid=10807526), [Takahashi N](http://www.ncbi.nlm.nih.gov/pubmed/?term=Takahashi N%5BAuthor%5D&cauthor=true&cauthor_uid=10807526), [Ashida T](http://www.ncbi.nlm.nih.gov/pubmed/?term=Ashida T%5BAuthor%5D&cauthor=true&cauthor_uid=10807526), [Nagai R](http://www.ncbi.nlm.nih.gov/pubmed/?term=Nagai R%5BAuthor%5D&cauthor=true&cauthor_uid=10807526): Serum Levels of Vascular Endothelial Growth Factor and Transforming Growth Factor-β1 in Patients with Atrial Fibrillation Undergoing

Defibrillation Therapy. [Jpn Heart J.](http://www.ncbi.nlm.nih.gov/pubmed/?term=Serum+Levels+of+Vascular+Endothelial+Growth+Factor+and+Transforming+Growth+Factor-β1+in+Patients+with+Atrial+Fibrillation+Undergoing+Defibrillation+Therapy) 2000 Jan;41(1):27-32.

**1 had no control group**

[Girerd N](http://www.ncbi.nlm.nih.gov/pubmed/?term=Girerd N%5BAuthor%5D&cauthor=true&cauthor_uid=24143210), [Scridon A](http://www.ncbi.nlm.nih.gov/pubmed/?term=Scridon A%5BAuthor%5D&cauthor=true&cauthor_uid=24143210), [Bessière F](http://www.ncbi.nlm.nih.gov/pubmed/?term=Bessière F%5BAuthor%5D&cauthor=true&cauthor_uid=24143210), [Chauveau S](http://www.ncbi.nlm.nih.gov/pubmed/?term=Chauveau S%5BAuthor%5D&cauthor=true&cauthor_uid=24143210), [Geloen A](http://www.ncbi.nlm.nih.gov/pubmed/?term=Geloen A%5BAuthor%5D&cauthor=true&cauthor_uid=24143210), [Boussel L](http://www.ncbi.nlm.nih.gov/pubmed/?term=Boussel L%5BAuthor%5D&cauthor=true&cauthor_uid=24143210), [Morel E](http://www.ncbi.nlm.nih.gov/pubmed/?term=Morel E%5BAuthor%5D&cauthor=true&cauthor_uid=24143210), [Chevalier P](http://www.ncbi.nlm.nih.gov/pubmed/?term=Chevalier P%5BAuthor%5D&cauthor=true&cauthor_uid=24143210): Periatrial Epicardial Fat Is Associated with Markers of Endothelial Dysfunction in Patients with Atrial Fibrillation. [PLoS One.](http://www.ncbi.nlm.nih.gov/pubmed/?term=Periatrial+Epicardial+Fat+Is+Associated+with+Markers+of+Endothelial+Dysfunction+in+Patients+with+Atrial+Fibrillation) 2013 Oct 15;8(10)

**1 had duplicated data**

Kim SK, Pak HN, Park JH, Ko KJ, Lee JS, Wi J, Choi JI, Kim YH: Serological Predictors for the Recurrence of Atrial Fibrillation After Electrical Cardioversion. Korean Circ J. 2010 Apr;40(4):185-90.

Duplicated with “Kim SK, Pak HN, Park JH, Ko KJ, Lee JS, Choi JI, Choi DH, Kim YH: [Clinical and serological predictors for the recurrence of atrial fibrillationafter electrical cardioversion.](http://www.ncbi.nlm.nih.gov/pubmed/19858160) Europace. 2009 Dec;11(12):1632-8.”
